# Letter from the Editor-in-Chief

**DOI:** 10.19102/icrm.2018.090208

**Published:** 2018-02-15

**Authors:** Moussa Mansour


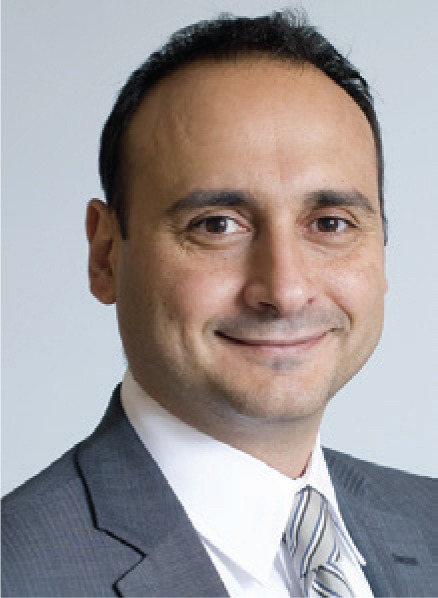


Dear Readers,

January is my most favorite month of the year because of the AF Symposium. This year’s meeting took place in Orlando between January 11 and 13, 2018 and was packed with highly educational presentations delivered by experts from around the world.

The Symposium opened with a series of lectures on the pathophysiology, risk factors, and genetics of atrial fibrillation (AF). New data on the role of aging, obesity, inflammation, and fibrosis were presented, including the important finding that epicardial fat volume is greater in patients with AF than in those without. Novel and exciting potential targets for treatment based on an improved understanding of the genetics of the disease were also discussed.

One of the highlights of the meeting this year was a half-a-day session on stroke prevention in AF. Topics included the role of screening for AF in stroke and a discussion of the most recent guidelines of the European Society of Cardiology, which have labeled the performance of a pulse check or ECG rhythm strip in patients who are older than 65 years of age as a class Ib indication. The controversy of whether anticoagulation can be stopped after AF ablation was also touched upon, with emerging evidence supporting stopping anticoagulation in selected patients when careful post-ablation monitoring is performed. Left atrial appendage (LAA) closure was extensively covered in this session by lectures and live case presentations on novel closure devices and new treatment strategies, including concomitant pulmonary vein (PV) isolation and LAA closure.

The second day of the meeting focused on ablation. The implications of fibrosis and myocardial fiber orientation on the formation of rotors and wave fractionation were discussed. New experimental data demonstrated that AF cycle length acceleration involves a cascade of short-lived rotors that form in rapid succession across both atria, with decreased rotor lifespan and transient acceleration occurring as AF stabilizes. That session was followed by a series of lectures and live case presentations focusing on mechanism-guided ablation. The role of non-PV drivers in persistent AF was covered extensively. Different AF mapping techniques appear to converge towards the identification of localized reentries in persistent patients. The role of the LAA in driving persistent AF continues to gain popularity: this year, two live case presentations demonstrated the use of cryo and radiofrequency energy (RF) for LAA isolation.

Emerging new technologies for AF ablation were covered in the second half of Day 2. The ongoing RADAR trial employing high-resolution contact mapping was described and appeared to present a promising approach to identify and treat drivers of persistent AF. Also, in this session, the results of the first-in-man AF-FICIENT I and RADIANCE trials were presented and showed that multielectrode RF balloons allow rapid and effective isolation of the PVs and may represent a promising alternative to focal ablation. By no means does this indicate a diminishing role of the focal ablation catheter, however. In fact, new focal catheters equipped with six temperature sensors located at the surface of the catheter were presented. They allow for accurate measurement of the catheter–tissue interface and permit the delivery of high-power, short-duration ablation guided by temperature sensing. A non-RF energy source, pulsed electric field ablation, was also introduced in this session as well. This form of energy may have important potential advantages including the formation of contiguous lesions, the sparing of non-cardiac tissue, and extremely fast delivery. Two additional novel technologies were illustrated in live case presentations: (1) the electroanatomical dielectric mapping system with the unique feature of lesion assessment and (2) the esophageal retraction balloon capable of deviating the esophagus safely and far away from the ablation field. For the second year, late breaking clinical trials were presented at the AF Symposium. The HEAT AF study showed that peak esophageal temperature measured using infrared thermography allowed for the prediction of esophageal thermal damage, while the REAFFIRM study showed that PV isolation plus rotor ablation was associated with a 75% success rate at 12 months, as compared with a 69% rate with PV isolation plus non-rotor conventional ablation. This difference did not reach statistical significance.

Day 3 of the meeting began with an update on clinical trials of new technologies in AF ablation and a summary of the 2017 Heart Rhythm Society/European Heart Rhythm Association/European Cardiac Arrhythmia Society/Asia Pacific Heart Rhythm Society/Latin American Society of Electrophysiology and Cardiac Stimulation consensus document on AF ablation. An informative lecture by representatives from the United States Food and Drug Administration provided helpful insights regarding the designing of clinical studies and the structure of the agency. High impact clinical trials were also presented in this session, including an update on the CABANA trial. Two important clinical studies, AATAC and CASTLE AF, were also covered, with both showing that catheter ablation for AF in heart failure improves patient arrhythmia burden, ejection fraction, rate of hospitalization, and survival. Another important development in AF ablation this year is the rising popularity of using high-power, short-duration RF delivery. At least two lectures in the meeting described very favorable results with the use of energy levels of 45 Watts (W) to 50 W for very short durations (5–15 seconds).

In summary, for its 23^rd^ consecutive year, this gathering of key opinion experts, business leaders, and practicing physicians culminated in a three-day packed event full of high-value educational lectures, live case presentations, and exhibitions of new technology from industry in a highly educational and interactive environment.

Sincerely,


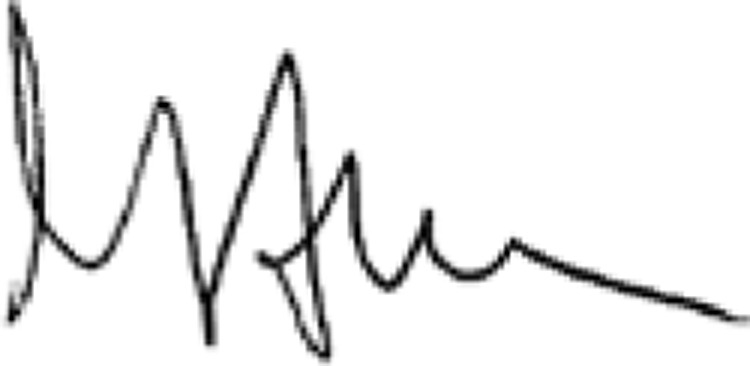


Moussa Mansour, md, fhrs, facc

Editor-in-Chief

The Journal of Innovations in Cardiac Rhythm Management

MMansour@InnovationsInCRM.com

Director, Atrial Fibrillation Program

Jeremy Ruskin and Dan Starks Endowed Chair in Cardiology

Massachusetts General Hospital

Boston, MA 02114

